# THE ART OF GRIEVING AMONG OLDER ADULTS ON TIKTOK

**DOI:** 10.1093/geroni/igae098.2567

**Published:** 2024-12-31

**Authors:** Reuben Ng, Nicole Indran

**Affiliations:** National University of Singapore, Singapore, Singapore; National University of Singapore, Singapore, Singapore

## Abstract

Although TikTok is dominated largely by younger persons, the platform has since gained traction among older adults, a number of whom have used it to cope with bereavement and grief. Presently, little is known about the therapeutic effects of creating TikTok content in the context of bereavement. However, there have been suggestions that the platform has the potential to serve as an accessible form of art therapy. This study explores how older adults mobilize audio-visual functions to communicate their experience of bereavement on TikTok. We compiled the most viewed videos of users aged 60 and above with at least 100,000 followers, generating 1,382 videos with over 3.5 billion views. Videos which did not feature older adults engaging in discourses on bereavement were excluded. This left us with 22 videos. Both inductive and deductive approaches guided the qualitative analysis. Three themes emerged: ‘Emotional Expression’ (Theme 1; 59%), ‘Maintaining a Relationship with the Deceased’ (Theme 2; 23%) and ‘Channelling Grief into Positive Emotions’ (Theme 3; 18%). This study represents a first step towards understanding the use of TikTok among older bereaved individuals. As findings from this study paint a rather optimistic portrait of the experience of uploading bereavement-related content on TikTok, future inquiry could dive into the potential harms of uploading such content on the application. In view of the enduring presence of TikTok, it is crucial to investigate the clinical effectiveness of using the platform as an art form to cope with bereavement.

